# Military Inhalational Exposures Outside the Theater of Conflict and Chronic Respiratory Symptoms

**DOI:** 10.1001/jamanetworkopen.2025.22080

**Published:** 2025-07-21

**Authors:** Reza Hosseini, Eric Garshick, Martin D. Slade, Andrew Timmons, Anna M. Korpak, Nicholas L. Smith, Karen S. Nakayama, Coleen P. Baird, Paul Ciminera, Farrah Kheradmand, Vincent S. Fan, Jaime E. Hart, Petros Koutrakis, Ware Glenn Kuschner, Octavian C. Ioachimescu, Michael Jerrett, Philippe R. Montgrain, Susan P. Proctor, Christine H. Wendt, Cherry Wongtrakool, Emily S. Wan, Paul D. Blanc, Carrie A. Redlich

**Affiliations:** 1 Occupational and Environmental Medicine, Department of Internal Medicine, Yale School of Medicine, New Haven, Connecticut; 2 Occupational and Environmental Medicine Section, Upstate Medical University, State University of New York, Syracuse; 3 Pulmonary, Allergy, Sleep and Critical Care Medicine Section, Medical Service, VA Boston Healthcare System, West Roxbury, Massachusetts; 4 Department of Medicine, Harvard Medical School, Boston, Massachusetts; 5 Seattle Epidemiologic Research and Information Center, Department of Veterans Affairs Office of Research and Development, VA Puget Sound Health Care System Seattle Division, Seattle, Washington; 6 Department of Epidemiology, University of Washington, Seattle; 7 Medical Science Affiliates, Columbia, Maryland; 8 Health Services Policy and Oversight, Office of the Assistant Secretary of Defense for Health Affairs, Washington, District of Columbia; 9 Department of Medicine, Michael E. DeBakey VA Medical Center, Houston, Texas; 10 Department of Medicine, Baylor College of Medicine, Houston, Texas; 11 VA Puget Sound Health Care System, Seattle, Washington; 12 Department of Medicine, University of Washington, Seattle; 13 Channing Division of Network Medicine, Brigham and Women’s Hospital, Boston, Massachusetts; 14 Department of Environmental Health, Harvard T.H. Chan School of Public Health, Boston, Massachusetts; 15 VA Palo Alto Health Care System, Palo Alto, California; 16 Stanford University School of Medicine, Stanford, California; 17 Clement J. Zablocki VA Medical Center, Milwaukee, Wisconsin; 18 Department of Medicine, Medical College of Wisconsin, Milwaukee; 19 Department of Environmental Health Sciences, Fielding School of Public Health, University of California, Los Angeles; 20 VA San Diego Health Care System, San Diego, California; 21 Department of Medicine, University of California, San Diego; 22 US Army Research Institute of Environmental Medicine, Natick, Massachusetts; 23 VA Research Service, VA Boston Healthcare System, Boston, Massachusetts; 24 Minneapolis VA Medical Center, Minneapolis, Minnesota; 25 Department of Medicine, University of Minnesota, Minneapolis; 26 Atlanta VA Healthcare System, Decatur, Georgia; 27 Department of Medicine, Emory University School of Medicine, Atlanta, Georgia; 28 San Francisco VA Health Care System, San Francisco, California; 29 Division of Occupational, Environmental, and Climate Medicine, Department of Medicine, University of California, San Francisco

## Abstract

**Importance:**

Deployment to Afghanistan and Southwest Asia has been associated with adverse respiratory health outcomes. However, the impact of inhalational exposures (eg, vapor, dust, gas, fumes), which are known correlates of reduced lung function and future chronic lung disease, during military service time outside this deployment period has not been assessed.

**Objective:**

To assess military inhalational exposures during nonwartime routine activities and their associations with chronic respiratory symptoms.

**Design, Setting, and Participants:**

This cross-sectional study used data from the US Department of Veterans Affairs Service and Health Among Deployed Veterans study. US veterans who served between October 7, 2001, and February 28, 2017; deployed to Afghanistan or Southwest Asia; and living near 6 Veterans Affairs sites were randomly selected from Defense Manpower Data Center records. Participants completed interviewer-administered multi-item questionnaires about 29 exposures related to active duty military service time when not deployed. Onsite visits occurred between April 27, 2018, and March 13, 2020, and analyses were performed between April 1, 2023, and February 10, 2025.

**Exposure:**

Inhalation exposures during active duty military service time.

**Main Outcomes and Measures:**

The main outcomes were chronic respiratory symptoms of dyspnea, wheeze in the previous 12 months, and chronic bronchitis. Using factor analysis, the 29 exposures were reduced to 20 items and categorized into 5 factors. Responses were scored ordinally (0, 1, 2) according to exposure prevalence and duration. Generalized linear modeling was used to explore associations between exposures and chronic respiratory symptoms.

**Results:**

The sample included 1712 veterans (median [IQR] age, 37 [33-45] years; 1522 male [88.9%]) who had military service other than during their deployment to Afghanistan or Southwest Asia. The median (IQR) total active duty military service duration was 77 (57-128) months, with 82.8% of their service time spent outside the theater of conflict. The prevalence of dyspnea, chronic bronchitis, and wheeze was 7.0% (117 participants), 7.1% (121 participants), and 15.2% (260 participants), respectively. The most commonly reported exposure categories were combustion and ground dust (1014 participants [59.2%]), aircraft maintenance (812 participants [47.4%]), and heavy equipment maintenance (783 participants [45.7%]). Adjusted multivariable analyses identified significant associations between heavy equipment maintenance exposures and dyspnea (odds ratio [OR], 1.33; 95% CI, 1.06-1.68) and wheeze (OR, 1.29; 95% CI, 1.10-1.52). Aircraft maintenance exposures were significantly associated with wheeze (OR, 1.22; 95% CI, 1.01-1.47). No statistically significant associations were found between these exposures and chronic bronchitis.

**Conclusions and Relevance:**

This cross-sectional study shows significant associations between heavy equipment maintenance and aircraft maintenance inhalation exposures outside of deployment with chronic respiratory symptoms among US veterans. These findings suggest that certain military inhalational exposures may contribute to the development of chronic lung disease and that policy interventions to reduce such exposures may protect the long-term respiratory health of military personnel.

## Key Points

**Question:**

Are inhalational exposures (eg, vapor, gas, dust, fumes) during nonwartime routine military activities associated with chronic respiratory symptoms?

**Findings:**

In this cross-sectional study of 1712 US veterans, inhalational exposures from heavy equipment and aircraft maintenance were common and significantly associated with respiratory symptoms, particularly dyspnea and wheeze.

**Meaning:**

These findings suggest an increased risk of chronic lung disease from specific frequent inhalational exposures during military service, highlighting an opportunity to mitigate risk.

## Teaser

This cross-sectional study assesses the association of inhalational exposures during nonwartime routine miltary activities with chronic respiratory symptoms among US veterans.

## Introduction

The deployment of military personnel to Southwest Asia and Afghanistan has shone a spotlight on concerns from inhalational exposures during deployment, especially from open-air solid waste incineration (eg, burn pits), regarding an increased risk of chronic lung disease.^1,2,3,4,5^ Clinical series and epidemiologic studies on military personnel with a history of deployment to Southwest Asia and Afghanistan have described a variety of respiratory symptoms and illnesses, including dyspnea, chronic cough, wheeze, asthma, bronchitis, sinusitis, and constrictive bronchiolitis.^6,7,8,9,10^ Chronic respiratory symptoms, such as dyspnea, persistent wheeze, and chronic cough, have been shown to correlate with chronic lung disease and reduced lung function, including obstructive and restrictive physiology.^11,12,13,14,15^

For US military personnel who have deployed to theaters of conflict, however, the majority of their total active duty service time typically is spent outside these arenas. These personnel engage in a range of activities that may entail potentially harmful inhalational exposures, such as maintenance of equipment and training exercises.^16^ In contrast to deployment-related exposures, whether inhalational exposures encountered during military service outside theaters of conflict are associated with adverse respiratory outcomes has received relatively scant attention. In this study, our aim was to characterize occupational and environmental inhalation exposures during military service outside the main theater of conflict among US veterans and investigate the hypothesis that such exposures may be associated with the development of chronic respiratory diseases as assessed by chronic respiratory symptoms.

## Methods

### Overview and Study Recruitment

This cross-sectional study used health and military service data from the US Department of Veterans Affairs’s (VA’s) Service and Health Among Deployed Veterans study. This study was approved by the VA Central Institutional Review Board, and all participants provided written informed consent. We used the Strengthening the Reporting of Observational Studies in Epidemiology (STROBE) reporting guidelines for cross-sectional studies.^17^

Participants completed onsite visits from April 27, 2018, to March 13, 2020. Analyses were performed from April 1, 2023, to February 10, 2025. Detailed methodology with regard to eligibility, study recruitment, interviewer-administered questionnaires, and the approach to exposure assessment has been delineated previously in Garshick et al.^18^ This prior study explored associations of inhalational exposures during military deployment to Southwest Asia and Afghanistan with chronic respiratory symptoms but did not examine nondeployment exposures.

### Definition of Deployment and Outside Theater of Conflict

Within the scope of this article, the term deployment refers specifically to land-based military assignment to Afghanistan, Kyrgyzstan, Iraq, Kuwait, Qatar, the United Arab Emirates, or Djibouti during a period when these deployments were in areas of conflict. Outside theater of conflict is defined as any active duty military service that did not involve deployment to these countries during that period, including time spent during military training, maintenance of equipment, or other nonwartime military service.

### Study Participants

Participants were chosen randomly from Defense Manpower Data Center deployment roster records. Recruitment was conducted through a combination of postal mail and telephone calls to 6913 eligible individuals, of whom 2299 (33.3%) agreed to participate. Of individuals agreeing to participate, 1967 completed the onsite visits between 2018 and 2020. Study size was not based on power calculations; rather, it represents participants enrolled during the initial phase of the parent study, which was interrupted by the COVID-19 pandemic. Veterans (including Reserve or National Guard members but not on active duty) were eligible for this study if they served in the Army, Air Force, or Marine Corps; had served on active duty between October 1, 2001, and February 28, 2017, with at least 1 deployment to 1 of the 7 aforementioned countries; had active duty military service time outside deployments to theaters of conflict; and lived within a 25-mile radius of 1 of the 6 participating VA medical centers across the US (Atlanta, Georgia; Boston, Massachusetts; Houston, Texas; Minneapolis, Minnesota; San Diego, California; and Seattle, Washington).

### Study Outcomes

Participants completed in-person visits that consisted of interviewer-administered structured questionnaires, including demographic (age, sex, self-reported race and ethnicity, educational level, body mass index [BMI], income, and marital status), smoking status, and military service–related characteristics (branch, rank, duration, and deployments), and a multi-item questionnaire to evaluate inhalational exposures. Self-reported race and ethnicity (American Indian or Alaska Native, Asian or Pacific Islander, Black or African American, White, multiracial, or other [Hispanic or Latino, Middle Eastern, Native Hawaiian, other]) were collected to describe the study population and assess potential differences in exposures or outcomes. Symptoms were assessed using items derived from the well-validated American Thoracic Society Division of Lung Diseases 1978 respiratory questionnaire^19^ and National Health and Nutrition Examination Survey respiratory health questionnaire.^20^ Symptoms of dyspnea were identified by a positive response to the question, “Other than with strenuous exercise, are you troubled with breathlessness,” and excluded respondents who used an assistive device for mobility. Wheeze was defined by any reported chest wheezing or whistling in the previous 12 months. Chronic bronchitis was defined by the report of both cough and phlegm for 3 consecutive months or more during the year and established how many of these had 2 or more consecutive years of such symptoms.

### Exposure Assessment

Detailed methodology for the exposure assessment and scoring applied to the active deployment period have been previously described by Garshick et al.^18^ The exposure questionnaire used in this study was part of the original study questionnaire; however, the data regarding nondeployment exposures had not been previously analyzed or reported. Respondents were asked, “After October 1, 2001, did you spend 1 month or more in total in a country other than Afghanistan, Kyrgyzstan, Iraq, Kuwait, Qatar, the United Arab Emirates, or Djibouti? This includes active duty time when deployed or stationed within the United States.” The exposure battery for military service outside conflict zones comprised 29 questions addressing self-reported occupational and environmental exposures experienced during heavy levels of exposures (13 questions) and frequent activities (16 questions) (eAppendix in Supplement 1). Heavy level of exposure was defined as a sustained or direct exposure in proximity to the source or an exposure that could be clearly sensed at the time, for example, through effects on the eyes, throat, or breathing. Frequent activities were defined as regularly performed duties. Participants were asked to report the number of months with heavy exposure or frequent activities and then to estimate the number of days in a typical month that the exposure or activity occurred.

### Exposure Categorization and Scoring

We adapted the previously described exposure grouping framework that characterized deployment-associated inhalational exposure sources.^18^ Using this framework, we categorized the exposures into 4 distinct a priori exposure domains: exhaust fumes; work-related vapors, gas, dust, or fumes (VGDF); dust; and burn pit or smoke. The details of each category are outlined in eTable 1 in Supplement 1.

**Figure 1.**
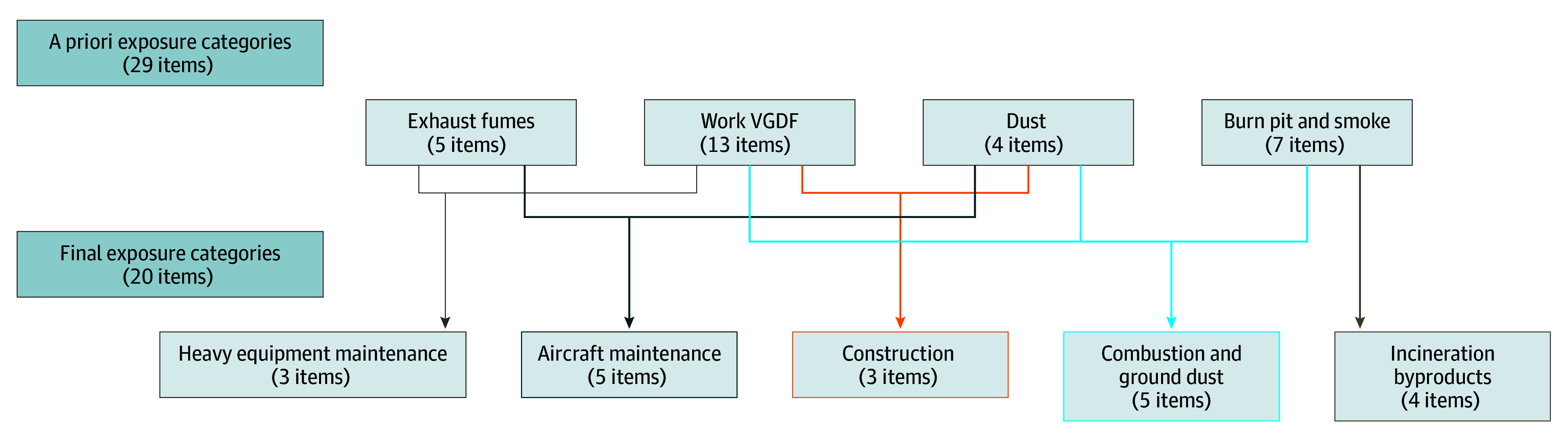
Summary of Factor Analyses The nondeployment questionnaire consisted of 29 questions and items. Nine items were removed due to low factor loading (eTable 2 in Supplement 1).

Figure 1 provides a graphic overview of the factor analysis. Given the large number of exposure variables, including overlapping exposures, we conducted a factor analysis to achieve more coherent modeling with the benefits of item reduction and fewer comparisons. An initial confirmatory factor analysis did not support the aforementioned a priori groupings of the 29 exposure items. Consequently, we used exploratory factor analysis to assess the underlying factor structure of the dataset. Based on the exploratory factor analysis, a 5-factor exposure model best captured the underlying correlations among the exposure items. Similar to the item reduction approach previously used,^18^ 9 survey exposure items with low factor loadings (<0.37) were removed from further analyses (eTable 2 in Supplement 1).

The analysis included 5 exposure categories (combustion and ground dust, aircraft maintenance, heavy equipment maintenance, construction, and incineration byproducts) with their corresponding 20 questionnaire items along with their prevalence and duration. For participants with any exposure, we quantified intensity based on the duration of occurrences expressed in person-days of exposure, which was converted to person-months by multiplying months exposed with days per month and dividing the product by 30. To calculate a summary score for each exposure category, we assigned a 3-level score to each exposure based on the median duration (0, no exposure; 1, moderate exposure [duration >0 but less than or equal to the median or any positive response without duration data]; ≥2, more frequent exposure [duration greater than the median]). We calculated summary scores in the 5 exposure categories by adding the values contributed by each respondent (0, 1, or 2) for each item in the category. We scaled each cumulative domain score to a possible maximum of 100 to standardize the scores among the exposure domains, which allowed for consistent comparisons across domains with different numbers of questions in each category. We categorized missing item responses (none exceeded 2.3%) as no, indicating no exposure. An ordinal score of level 1 was assigned to yes responses with missing intensity data.

### Statistical Analysis

All data analyses were conducted using SAS, version 9.4 (SAS Institute Inc).^21^ For descriptive characteristics, we report means and SDs for normally distributed variables and medians with IQRs for non–normally distributed variables. We used generalized linear mixed models capable of incorporating a binary response variable, with observations within each site considered as clusters, to investigate associations between respiratory outcomes (separate models for dyspnea, chronic bronchitis, and wheeze) and the 5 exposure categories. Final models were adjusted for potential confounders, including age, sex, race and ethnicity (White vs others), education (high school or less, some college, or bachelor’s degree or higher), income (less than $10 000 to $100 000 or more), marital status, BMI (calculated as weight in kilograms divided by height in meters squared) (<25 vs 25 to <30 and ≥30), smoking status (never vs former and current), and duration of deployment (as a continuous variable). We also adjusted for self-reported civilian workplace exposure to VGDF based on questionnaire responses and defined as at least 1 year of time spent in their primary full- or part-time civilian job with reported regular exposure to VGDF. Parameter confidence intervals were estimated using cluster bootstrapping on VA recruitment site to account for potential correlations among participants from the same site. Confidence intervals were obtained from the results of 1000 samples drawn with replacement. Odds ratios (ORs) and their 95% CIs were scaled to indicate a 20-unit change in estimated score, representing one-fifth (quintile) of a 100-point scaled score. A statistical significance level of α = .05 was used for all analyses.

## Results

Of the 1958 potential participants, 246 were excluded because they reported having no active duty military service outside deployment in the theater of conflict, leaving 1712 veterans included in the analysis (median [IQR] age, 37.4 [33.4-45.7] years; 190 female [11.1%] and 1522 male [88.9%]; 8 self-identified as American Indian or Alaska Native [0.5%], 80 as Asian [4.7%], 233 as Black [13.6%], 1180 as White [68.9%], and 199 as multiracial or other [11.6%] race and ethnicity). Characteristics of the study participants are summarized in Table 1 and eTable 3 in Supplement 1. Obesity was common (756 participants with BMI >30 [44.2%]), and 230 (13.4%) reported current and 549 (32.1%) former smoking status. In terms of service branches, 1017 participants (59.4%) served in the Army, 401 (23.4%) in the Marine Corps, and 269 (15.7%) in the Air Force.

**Table 1.** Participant Characteristics (N = 1712)

Characteristic^a^	Participants, No. (%)
Age, median (IQR), y	37.4 (33.4-45.7)
Sex	
Female	190 (11.1)
Male	1522 (88.9)
Race and ethnicity	
American Indian or Alaska Native	8 (0.5)
Asian or Pacific Islander	80 (4.7)
Black or African American	233 (13.6)
White	1180 (68.9)
Multiracial or other^b^	199 (11.6)
Educational level	
Less than high school or GED	156 (9.2)
Some college	394 (23.0)
Associate’s degree	284 (16.6)
Bachelor’s degree	536 (31.3)
Master’s degree or higher	341 (19.9)
BMI	
<25	250 (14.6)
25-30	704 (41.1)
>30	756 (44.2)
Smoking status	
Never	932 (54.4)
Former	549 (32.1)
Current	230 (13.4)
Military service	
Army	1017 (59.4)
Air Force	269 (15.7)
Marine Corps	401 (23.4)
Mixed or other^c^	23 (1.3)
Civilian job with VGDF	506 (29.6)
Service and deployment history, median (IQR)	
Total active duty military time, mo	77 (57-128)
Time since last deployment, mo	120 (91-157)
Time since separation, mo	62 (30-107)
No. of deployments	1 (1-2)
Deployment duration, mo	12 (7-17)
Respiratory symptoms	
Wheeze	260 (15.2)
Dyspnea	117 (7.0)
Chronic bronchitis	121 (7.1)

Abbreviations: BMI, body mass index (calculated as weight in kilograms divided by height in meters squared); GED, General Educational Development; VGDF, vapor, gas, dust, or fumes.

^a^ The number of participants with missing values were 12 for race and ethnicity, 1 for educational level, 2 for BMI, 2 for military branch, 1 for smoking status, and 40 for dyspnea.

^b^ Other race and ethnicity included Afro-Latino; Cape Verdean; Central American; Choctaw Indian; Dominican and Bulgarian; European; Hawaiian; Hispanic; Hispanic and Southern European; Italian; Italian, German, and Ecuadorian; Latino; Mexican American; Middle Eastern; Peruvian; Puerto Rican; and Taino Indian.

^c^ Other military service included Coast Guard and Navy.

The median total active duty military service duration was 77 months (IQR, 57-128 months), with a median of 63 months (IQR, 48-112 months) outside the theater of conflict. Most participants reported a single deployment, with a median total deployment duration of 12 months (IQR, 7-17 months). Thus, 82.8% of their active duty military service time was spent outside the theater of conflict. Interviews occurred a median of 10.0 years (IQR, 7.6-13.1 years) following the end of deployment to the theater of conflict and 5.2 years (IQR, 2.5-8.9 years) since separation from military service (including National Guard or Reserve duty). The prevalence of wheeze in the past 12 months, chronic bronchitis, and dyspnea was 15.2% (260 participants), 7.1% (121 participants), and 7.0% (117 participants), respectively.

The prevalence and duration of different exposures reported across the 5 exposure categories identified by the exploratory factor analysis are summarized in Table 2. The most commonly reported exposure categories were combustion and ground dust (1014 participants [59.2%] endorsing exposure to ≥1 item), aircraft maintenance (812 participants [47.4%]), and heavy equipment maintenance (783 participants [45.7%]). Exposure to items in either the construction or incineration byproducts categories was less common (213 participants [12.4%] and 123 participants [7.2%], respectively). The median duration of exposures ranged from 0.47 person-months (IQR, 0.13-2.67 person-months) for heavy direct exposure to smoke or fumes from burning vehicles or fires caused by explosives or other weapons to 20.27 person-months (IQR, 6.00-40.00 person-months) for maintenance of aircraft.

**Table 2.** Prevalence and Duration of the Exposures During Active Duty Military Service Outside Theater of Conflict (N = 1712)^a^

Exposure category with related items	Prevalence, No. (%)	Duration of exposure, median (IQR), person-months
**Combustion and ground dust (n = 1014 [59.2%])**		
Heavy direct exposure to smoke or fumes from burning vehicles or fires caused by explosives or other weapons	147 (8.6)	0.47 (0.13-2.67)
Heavy direct exposure to flares, marker smoke, smoke bombs, or other similar sources	486 (28.4)	0.67 (0.20-2.00)
Heavy dust from directly participating in convoy or other vehicle operations lasting ≥6 h in a row	370 (21.6)	1.00 (0.27-2.67)
Heavy dust generated by any other vehicle, aircraft, or equipment operations	454 (26.5)	1.55 (0.47-4.40)
Applying pesticide, insecticide, or repellent to your skin or to your own uniform	574 (33.5)	2.00 (0.53-6.00)
**Aircraft maintenance (n = 812 [47.4%])**		
Maintenance of aircraft	146 (8.5)	20.27 (6.00-40.00)
Use of solvents, lacquer, adhesives, or paint	437 (25.5)	2.00 (0.46-12.00)
Heavy sustained exposure from proximity to fixed-wing aircraft engine exhaust fumes	322 (18.8)	3.00 (0.67-22.67)
Heavy sustained exposure from proximity to helicopter engine exhaust fumes	365 (21.3)	1.60 (0.27-9.00)
Experience of any days when missions were halted due to poor air quality: no-fly days or days when operations were halted due to poor visibility	229 (13.4)	5.00 (2.00-15.00)
**Heavy equipment maintenance (n = 783 [45.7%])**		
Mechanical maintenance of ground vehicles	550 (32.1)	5.00 (1.60-14.00)
Maintenance of other machinery or heavy equipment	185 (10.8)	4.13 (1.20-15.40)
Heavy sustained exposure from proximity to generator engine exhaust fumes	419 (24.5)	2.83 (0.93-10.00)
**Construction (n = 213 [12.4%])**		
Building or other fixed structure construction	72 (4.2)	2.00 (0.50-4.00)
Work with exposure to plywood dusts or fumes	134 (7.8)	1.20 (0.33-4.00)
Road building or earth moving	91 (5.3)	1.40 (0.40-3.50)
**Incineration byproducts (n = 123 [7.2%])**		
Heavy exposure to smoke or fumes from personally operating or working with trash incineration or at a burn pit, including regularly burning trash and burn pit security	39 (2.3)	0.50 (0.23-2.57)
Heavy sustained exposure outdoors to smoke or fumes from burn pits or planned incineration, eg, when the wind changed or there was other heavy exposure	89 (5.2)	1.06 (0.40-4.00)
Heavy sustained exposure to smoke or fumes from burn pits or planned incineration coming into worksite or housing	60 (3.5)	1.33 (0.46-6.00)
Doing regular exercise or other physical exertion alongside the perimeter or right beside a burn pit site	38 (2.2)	3.50 (1.50-7.00)

^a^ Five exposure categories after October 1, 2001, were retained after exploratory factor analysis.

**Figure 2.**
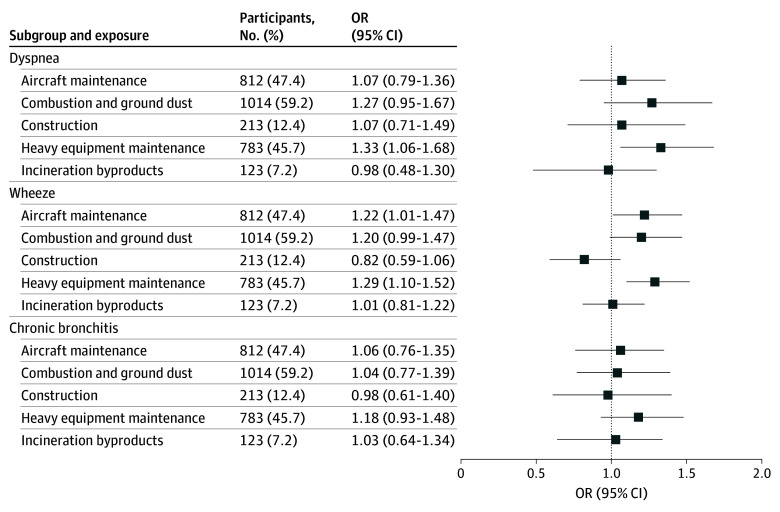
Adjusted Multivariable Models of Associations Between Exposure Groups (Percent Exposed) and Respiratory Symptoms (Percent Prevalence) Adjusted for age; sex; race and ethnicity; education; income; marital status; body mass index; smoking status; duration of deployment; and self-reported civilian workplace exposure to vapors, gas, dust, or fumes.

Results from the adjusted multivariable analyses between each exposure category and respiratory symptoms, scaled per a 20-unit change in exposure score, are shown in Table 3 and Figure 2. Unadjusted model results are provided in eTable 4 in Supplement 1. Exposure to heavy equipment maintenance was significantly associated with an increased odds of dyspnea (OR, 1.33; 95% CI, 1.06-1.68) and wheeze (OR, 1.29; 95% CI, 1.10-1.52). Exposure to aircraft maintenance was significantly associated with wheeze (OR, 1.22; 95% CI, 1.01-1.47). The odds were increased but not significant for wheeze from combustion and ground dust exposure (OR, 1.20; 95% CI, 0.99-1.47) and dyspnea (OR, 1.27; 95% CI, 0.95-1.67). Incineration byproducts and construction exposure categories were not associated with any of the chronic respiratory symptoms. None of the exposure categories showed an association with chronic bronchitis. These analyses were adjusted for age, sex, race and ethnicity, income, marital status, education, BMI, smoking status, civilian VGDF exposure, and deployment duration. The adjusted model results for socioeconomic covariates are presented in eTable 5 in Supplement 1. Longer deployment duration did not show an association with chronic respiratory symptoms. Among other covariates, BMI greater than 30 was associated with a significantly increased odds of dyspnea (OR, 3.49; 95% CI, 1.89-10.74), wheeze (OR, 2.12; 95% CI, 1.40-3.55), and chronic bronchitis (OR, 2.82; 95% CI, 1.51-8.02), and current smoking was associated with a significantly increased odds of wheeze (OR, 2.06; 95% CI, 1.36-3.22) and chronic bronchitis (OR, 2.49; 95% CI, 1.45-4.44).

**Table 3.** Association of Exposure Factors With Symptoms (N = 1712)

Exposure category	Participants, No. (%)	Symptoms, OR (95% CI)		
		Dyspnea (n = 117 of 1672 [7.0%])	Wheeze (n = 260 of 1712 [15.2%])^a^	Chronic bronchitis (n = 121 of 1712 [7.1%])^a^
Combustion and ground dust	1014 (59.2)	1.27 (0.95-1.67)	1.20 (0.99-1.47)	1.04 (0.77-1.39)
Aircraft maintenance	812 (47.4)	1.07 (0.79-1.36)	1.22 (1.01-1.47)	1.06 (0.76-1.35)
Heavy equipment maintenance	783 (45.7)	1.33 (1.06-1.68)	1.29 (1.10-1.52)	1.18 (0.93-1.48)
Construction	213 (12.4)	1.07 (0.71-1.49)	0.82 (0.59-1.06)	0.98 (0.61-1.40)
Incineration byproducts	123 (7.2)	0.98 (0.48-1.30)	1.01 (0.81-1.22)	1.03 (0.64-1.34)
Covariates				
Age	NA	1.03 (0.78-1.29)	1.22 (1.04-1.43)	1.24 (1.03-1.55)
Female sex	190 (11.1)	1.76 (0.87-3.22)	1.30 (0.74-2.08)	1.48 (0.73-2.56)
BMI 25-30	704 (41.1)	2.33 (1.12-7.48)	1.07 (0.67-1.81)	1.37 (0.69-3.67)
BMI >30	756 (44.2)	3.49 (1.89-10.74)	2.12 (1.40-3.55)	2.82 (1.51-8.02)
Former smoker	549 (32.1)	1.50 (0.92-2.35)	1.25 (0.87-1.73)	1.16 (0.69-1.78)
Current smoker	230 (13.4)	1.71 (0.97-2.94)	2.06 (1.36-3.22)	2.49 (1.45-4.44)
Deployment duration	NA	1.14 (0.87-1.48)	0.98 (0.79-1.19)	1.20 (0.91-1.57)

Abbreviations: BMI, body mass index (calculated as weight in kilograms divided by height in meters squared); NA, not applicable; OR, odds ratio.

^a^ Adjusted for age; sex; BMI (reference, <25), smoking status (reference, never), and deployment duration. Odds ratios for exposure factor scores and associations with each symptom scaled per 20-unit change in exposure score, OR for age expressed per 10 years, and OR for deployment duration expressed per 1 year.

## Discussion

In this cross-sectional study, we characterized occupational and environmental inhalation exposures experienced by US military personnel during their service outside the theater of conflict using a multi-item exposure questionnaire. Our results show a significant association between chronic respiratory symptoms in military personnel and certain inhalational exposures during their service outside the principal theater of conflict during their active military duty. Aircraft maintenance and heavy equipment maintenance exposures were significantly associated with an increased odds of dyspnea and wheeze in this cohort.

This study focused on the impact of military inhalational exposures outside the theater of conflict, which is highly relevant as military personnel represent a large workforce worldwide, with an estimated 18.7 million active or retired military personnel in the US (approximately 7.2% of the adult population and 14.0% of adult men).^22^ Even among service members deployed to conflict zones, most of their military service time is spent outside theaters of conflict. In this study, which recruited former military personnel deployed to Afghanistan and Southwest Asia, more than 83% of the participants’ total military service time was spent outside this theater. In addition to training exercises, routine military activities commonly involve the operation, maintenance, and repair of various heavy equipment and weapons, such as tanks, transport vehicles, aircraft, and various weapon systems. Such activities entail numerous inhalational exposures, such as exhaust fumes, welding fumes, combustion products, construction dusts, and solvents.

To date, studies have primarily focused on inhalational exposures related to wartime deployment, with an emphasis on burn pits.^1,2,3,5,16,18^ Using a similar methodological approach to this study, Garshick et al^18^ identified 4 other major deployment exposure categories (military job-related VGDF, ground dust and engine exhaust, other combustion byproducts, and toxicants) in addition to burn pit smoke and found an increased odds of chronic respiratory symptoms among individuals with exposure to burn pit smoke and VGDF. However, these studies did not consider inhalational exposures during nondeployment military service time.

Our findings that inhalational exposures from aircraft and heavy equipment maintenance are associated with chronic respiratory symptoms are consistent with a large body of literature showing that job-related VGDF exposures in various other settings (eg, construction, agriculture, industrial manufacturing, mining) are significant risk factors for chronic lung disease.^18,23,24,25,26,27^ Our findings are also consistent with the more limited literature showing that former military personnel have an increased risk of chronic obstructive pulmonary disease compared with the general population, taking into consideration smoking.^28,29,30^ However, these studies have provided limited characterization of exposures. In contrast, our analysis characterized the types, duration, and magnitude of inhalational exposures during a wide spectrum of routine military service activities.

### Strengths and Limitations

There are several important strengths to this study. Our multi-item battery of exposure questions followed an iterative process involving factor analysis and systematic item reduction to define 5 distinct exposure categories. This strategy allowed us to identify patterns within the data rather than impose assumptions. The resulting exposure domains were closely aligned with recognizable occupational activities of military service members, supporting their face validity. We used the multi-item battery to capture a wide range of inhalational exposures and their durations and provided a standard definition of exposure intensity. This approach enabled characterization of exposures beyond a simple present-or-not dichotomy. Additionally, we used factor analysis to examine the underlying structure of the data without imposing strict assumptions about associations between variables. Of note, the factor analysis using nondeployment-reported exposures identified 5 coherent exposure groupings that differed distinctly from the 5 deployment-related inhalational exposure categories and their prevalences identified by Garshick et al.^18^ For example, in Garshick et al, 72.7% of participants reported exposure to burn pit smoke vs 7.2% observed in our study. These differences suggest that non–theater-of-conflict military inhalational exposures differ in their interrelationships compared with deployment-related inhalational exposures.

Another strength of the study is that the veteran participants were recruited randomly, not selected or self-referred based on their symptoms or concerns regarding prior military exposures, reducing the likelihood of selection and reporting biases. As previously noted, the use of a multi-item battery of questions spanning a range of potential exposures and a systematic item reduction strategy reduced the likelihood of reporting bias. In this study, we did not find any association with incineration byproducts (eg, burn pits), which may have been influenced by low prevalence (7.2%) and short duration of such exposures (0.5-3.5 person-months). The lack of association of chronic respiratory symptoms with prior identified exposures of concern, as well the association of different exposure categories with different respiratory symptoms, also argue against a general bias toward overreporting of inhalational exposures. Our findings also revealed that, as expected, covariates such as increasing age, smoking, and BMI >25 were associated with an increased odds of chronic respiratory symptoms, as seen in other studies,^31,32,33,34^ increasing confidence in the validity of the questionnaire data obtained. While we did not observe an association between smoking and dyspnea, there was an elevated OR for current and former smoking, consistent with literature that smoking is a risk factor for dyspnea. This finding suggests that the relatively young age of participants may have contributed to a healthy worker effect of serving in the military.

There are also several limitations to our study that should be considered. The study outcomes were based on symptoms rather than diagnostic outcomes or objective test results. Although, spirometrically defined outcomes are a part of the parent study, these will not be available for analysis until termination of data collection. Additionally, radiographic imaging was not obtained, and information on medication usage and comorbid conditions was not available for this analysis. However, chronic respiratory symptoms are strong indicators of reduced lung function and chronic lung disease.^11,12,13,14,15,35^ The respiratory questionnaires used in this study are well-validated for assessing respiratory health due to their high internal consistency and correlation with objective measures of pulmonary function.^36,37^ In addition, the prevalence of chronic respiratory symptoms reported is consistent with other studies that have documented a prevalence of 7% to 16% among former military personnel previously deployed to Afghanistan and Southwest Asia.^18,38,39^ The questionnaires primarily focused on respiratory symptoms rather than self-reported diagnoses. Although limited survey data were collected on medical diagnoses, these were outside the scope of our analysis. Another limitation was that the study participants were recruited from the vicinity of a select number of VA facilities and may not be representative of the broader veteran population.

There are also limitations to the exposure assessment. Quantitative exposure data, such as industrial hygiene measurements, were not available for the numerous military inhalational exposures encountered. However, the questionnaire data enabled semiquantitative assessment of the exposures by defining heavy exposure and determining the duration of each exposure. Given that as the exact timing of exposures within the duration of military service was not available, we were unable to model lag effects. While self-reported exposure data are another concern, several factors suggested that selective recall may not fully explain the findings. Notably, exposures with the greatest historical attention (eg, burn pits) had relatively low reported prevalence and little association with symptoms, and participants were randomly recruited rather than self-referred, reducing the likelihood of such bias.

This analysis did not fully account for specific deployment-related and civilian work exposures, which may also contribute to chronic respiratory symptoms, even though the time spent deployed to Afghanistan and Southwest Asia accounted for less than 18% of total service time. To address these concerns, our analysis adjusted for deployment duration and self-reported civilian occupational exposure to VGDF and found that neither factor was significantly associated with chronic respiratory symptoms. We recognize that inhalational exposures during deployment may have influenced nondeployment exposure status; however, military personnel typically have limited choice over their duty assignments, reducing the potential for confounding by indication. Our questionnaires were not designed to assess pre-2001 military exposures, so it is possible that we underestimated military exposures outside theaters of conflict. Finally, we were unable to account for ambient exposures to particulate air pollution, which also may have contributed to chronic respiratory symptoms. However, including study site as a fixed rather than random variable yielded similar findings, suggesting that environmental conditions (eg, air pollution) did not influence the observed associations. Future studies should consider the contribution of these different sources of inhalational exposures to chronic respiratory disease in veterans.

## Conclusions

This cross-sectional study shows that military occupational exposures from heavy equipment maintenance and aircraft maintenance are associated with an increased odds of chronic respiratory symptoms that may lead to the development of chronic lung disease. Although further research is needed, consistent with the precautionary principle, our findings suggest that policy interventions to reduce certain inhalational exposures may improve the long-term respiratory health of military personnel. For individual patients, clinicians should inquire about inhalational exposures during military service as part of their assessment of risk factors for the development of chronic lung disease.

## Notes

Supplement 1. **eAppendix.** CSP #595 Exposure Module: Active Duty Military Time Outside of Deployments to Afghanistan and Southwest Asia **eTable 1.** A Priori Exposure Categories **eTable 2.** Questionnaire Items Excluded Due to Low Factor Loading (<0.37) or Loading Across Multiple Factor Groups **eTable 3.** Additional Participant Characteristics **eTable 4.** Unadjusted (Crude) Odds Ratios for Respiratory Symptoms by Exposure Domain **eTable 5.** Adjusted Odds Ratios for Socioeconomic Covariates and Respiratory Symptoms

Supplement 2.
Data Sharing Statement
