# Supplementary material for: Military Inhalational Exposures Outside the Theater of Conflict and Chronic Respiratory Symptoms
Source: JAMA Netw Open. 2025 Jul 21;8(7):e2522080. doi: 10.1001/jamanetworkopen.2025.22080 (PMC12281241; doi:10.1001/jamanetworkopen.2025.22080)
Supplement: Supplement 1. — eAppendix. CSP #595 Exposure Module: Active Duty Military Time Outside of Deployments to Afghanistan and Southwest Asia eTable 1. A Priori Exposure Categories eTable 2. Questionnaire Items Excluded Due to Low Factor Loading (<0.37) or Loading Across Multiple Factor Groups eTable 3. Additional Participant Characteristics eTable 4. Unadjusted (Crude) Odds Ratios for Respiratory Symptoms by Exposure Domain eTable 5. Adjusted Odds Ratios for Socioeconomic Covariates and Respiratory Symptoms [file jamanetwopen-e2522080-s001.pdf]

Supplemental Online Content

Hosseini R, Garshick E, Slade MD, et al. Military inhalational exposures outside the theater of conflict and chronic respiratory symptoms. *JAMA Netw Open*. 2025;8(7):e2522080.
doi:10.1001/jamanetworkopen.2025.22080

eAppendix. CSP #595 Exposure Module: Active Duty Military Time Outside of Deployments to Afghanistan and Southwest Asia

eTable 1. A Priori Exposure Categories

eTable 2. Questionnaire Items Excluded Due to Low Factor Loading (<0.37) or Loading Across Multiple Factor Groups

eTable 3. Additional Participant Characteristics

eTable 4. Unadjusted (Crude) Odds Ratios for Respiratory Symptoms by Exposure Domain

eTable 5. Adjusted Odds Ratios for Socioeconomic Covariates and Respiratory Symptoms

This supplemental material has been provided by the authors to give readers additional information about their work.

© 2025 Hosseini R et al. *JAMA Network Open*.

eAppendix. CSP #595 Exposure Module: Active Duty Military Time Outside of Deployments to the Afghanistan and Southwest Asia

Thinking about your active-duty military time after October 1, 2001, did you spend one month or more in total in a country **other than** Afghanistan, Kyrgyzstan, Iraq, Kuwait, Qatar, the United Arab Emirates, or Djibouti? This includes active-duty time when deployed or stationed within the United States. **[PROMPT IF NEEDED: THIS INCLUDES DEPLOYMENTS, MISSIONS, TRAINING, AS WELL AS PERIODS OF NATIONAL GUARD OR RESERVE SERVICE]**

☐ Yes ⇨ **START WITH QUESTION 1**
☐ No

**The following questions ask about "heavy" levels of exposure. Heavy means a sustained or direct exposure in close proximity - exposure that you could clearly sense at the time, for example, through effects on the eyes, throat, or breathing. Did you experience:**		**Don't Know**	**No**	**Yes**	**Approximately, for how many months did this exposure occur? If this occurred during 1 month or less, say 1 month.** **[ENTER 1 IF EXPOSURE OCURRED FOR ≤ 1 MONTH]**	**[ASK IF EXPOSURE OCCURRED FOR ≤ 1 MONTH]** **On approximately how many days did this exposure occur?**	**[ASK IF EXPOSURE OCCURRED FOR >1 MONTH]** **In a typical month when you had this exposure, on approximately how many days did this occur?**
1.	Heavy exposure to smoke or fumes from personally operating or working with trash incineration or at a burn pit? This includes regularly burning trash and burn pit security.	☐	☐	☐ ⇨	☐☐☐	☐☐☐	☐☐☐
2.	Heavy sustained exposure outdoors to smoke or fumes from burn pits or planned incineration, for example when the wind changed or there was other heavy exposure?	☐	☐	☐ ⇨	☐☐☐	☐☐☐	☐☐☐
3.	Heavy sustained exposure to smoke or fumes from burn pits or planned incineration coming into your work site or housing?	☐	☐	☐ ⇨	☐☐☐	☐☐☐	☐☐☐

4.	Heavy direct exposure to smoke or fumes from burning vehicles or fires caused by explosives or other weapons?	☐	☐	☐ ⇨	☐☐☐	☐☐☐	☐☐☐
5.	Heavy direct exposure to flares, marker smoke, smoke bombs, or other similar sources?	☐	☐	☐ ⇨	☐☐☐	☐☐☐	☐☐☐
6.	Other heavy exposure to smoke or fumes from other burning or open combustion sources not previously mentioned? Do not count engine exhaust.	☐	☐	☐ ⇨	☐☐☐	☐☐☐	☐☐☐
7.	Heavy dust from directly participating in convoy or other vehicle operations lasting 6 hours or more in a row?	☐	☐	☐ ⇨	☐☐☐	☐☐☐	☐☐☐
8.	Heavy dust generated by any other vehicle, aircraft, or equipment operations?	☐	☐	☐ ⇨	☐☐☐	☐☐☐	☐☐☐
9.	Heavy sustained exposure from close proximity to fixed wing aircraft engine exhaust fumes?	☐	☐	☐ ⇨	☐☐☐	☐☐☐	☐☐☐
10.	Heavy sustained exposure from close proximity to helicopter engine exhaust fumes?	☐	☐	☐ ⇨	☐☐☐	☐☐☐	☐☐☐
11.	Heavy sustained exposure from close proximity to truck, Humvee, tank, APC, or Stryker engine exhaust fumes?	☐	☐	☐ ⇨	☐☐☐	☐☐☐	☐☐☐
12.	Heavy sustained exposure from close proximity to generator engine exhaust fumes?	☐	☐	☐ ⇨	☐☐☐	☐☐☐	☐☐☐
13.	Heavy exposure to any other engine or compressor exhaust fumes?	☐	☐	☐ ⇨	☐☐☐	☐☐☐	☐☐☐

© 2025 Hosseini R et al. *JAMA Network Open*.

The following questions concern potential ongoing or frequent activities. Did you regularly perform any of the following activities? [PROMPT: when NOT deployed to Afghanistan, Kyrgyzstan, Iraq, Kuwait, Qatar, the United Arab Emirates, or Djibouti]		Don't Know	No	Yes	Approximately, for how many months did this occur? If this occurred during 1 month or less, say 1 month. [ENTER 1 IF OCURRED FOR ≤ 1 MONTH]	ASK IF OCCURRED FOR ≤ 1 MONTH] On approximately how many days did this occur	ASK IF OCCURRED FOR >1 MONTH] In a typical month, on approximately how many days did this occur?
14.	Doing regular exercise or other physical exertion alongside of the perimeter or right beside a burn pit site?	☐	☐	☐ ⇨	☐☐☐	☐☐☐	☐☐☐
15.	Vehicle, aircraft, generator, or other engine refueling operations?	☐	☐	☐ ⇨	☐☐☐	☐☐☐	☐☐☐
16.	Mechanical maintenance of ground vehicles?	☐	☐	☐ ⇨	☐☐☐	☐☐☐	☐☐☐
17.	Maintenance of aircraft?	☐	☐	☐ ⇨	☐☐☐	☐☐☐	☐☐☐
18.	Maintenance of other machinery or heavy equipment?	☐	☐	☐ ⇨	☐☐☐	☐☐☐	☐☐☐
19.	Road building or earth moving?	☐	☐	☐ ⇨	☐☐☐	☐☐☐	☐☐☐
20.	Building or other fixed structure construction?	☐	☐	☐ ⇨	☐☐☐	☐☐☐	☐☐☐
21.	Welding or flame cutting?	☐	☐	☐ ⇨	☐☐☐	☐☐☐	☐☐☐
22.	Use of asbestos?	☐	☐	☐ ⇨	☐☐☐	☐☐☐	☐☐☐
23.	Use of fiberglass or insulation material other than asbestos?	☐	☐	☐ ⇨	☐☐☐	☐☐☐	☐☐☐
24.	Use of solvents, lacquer, adhesives, or paint?	☐	☐	☐ ⇨	☐☐☐	☐☐☐	☐☐☐

© 2025 Hosseini R et al. *JAMA Network Open*.

25.	Work with exposure to plywood dusts or fumes?	☐	☐	☐ ⇨	☐☐☐	☐☐☐	☐☐☐
26.	Applying pesticide, insecticide, or repellent to your skin or to your own uniform?	☐	☐	☐ ⇨	☐☐☐	☐☐☐	☐☐☐
27.	Other pesticide, insecticide, or repellent application or handling?	☐	☐	☐ ⇨	☐☐☐	☐☐☐	☐☐☐
28.	As a part of your duties was there any other regular heavy exposure to vapors, gas, dust, or fumes not already covered?	☐	☐	☐ ⇨	☐☐☐	☐☐☐	☐☐☐
	[If yes:] Specify exposure ________________________________						

**The final question concerns “no fly days”.**		**Don’t [illegible]**	**No**	**Yes**	**How many times did this occur?**
29.	Did you experience any days when missions were halted due to poor air quality: “no fly days,” or days when operations were halted due to poor visibility?	☐	☐	☐ ⇨	☐☐☐

© 2025 Hosseini R et al. *JAMA Network Open*.

eTable 1. A Priori Exposure Categories

Exposure Category	Sources of Exposure
**Exhaust Fumes (5 Items)**	1. Heavy sustained exposure from close proximity to fixed wing aircraft engine exhaust fumes?<br>2. Heavy sustained exposure from close proximity to helicopter engine exhaust fumes?<br>3. Heavy sustained exposure from close proximity to truck, Humvee, tank, APC, or Stryker engine exhaust fumes?<br>4. Heavy sustained exposure from close proximity to generator engine exhaust fumes?<br>5. Heavy exposure to any other engine or compressor exhaust fumes?
**Work VGDF (13 Items)**	1. Mechanical maintenance of ground vehicles?<br>2. Maintenance of aircraft?<br>3. Maintenance of other machinery or heavy equipment?<br>4. Building or other fixed structure construction?<br>5. Welding or flame cutting?<br>6. Vehicle, aircraft, generator, or other engine refueling operations?<br>7. Use of asbestos?<br>8. Use of fiberglass or insulation material other than asbestos?<br>9. Use of solvents, lacquer, adhesives, or paint?<br>10. Work with exposure to plywood dusts or fumes?<br>11. Applying pesticide, insecticide, or repellent to your skin or to your own uniform?<br>12. Other pesticide, insecticide, or repellent application or handling?<br>13. As a part of your duties was there any other regular heavy exposure to vapors, gas, dust, or fumes not already covered?
**Dust (4 Items)**	1. Heavy dust from directly participating in convoy or other vehicle operations lasting 6 hours or more in a row?<br>2. Heavy dust generated by any other vehicle, aircraft, or equipment operations?<br>3. Road building or earth moving?<br>4. Did you experience any days when missions were halted due to poor air quality: “no fly days,” or days when operations were halted due to poor visibility?
**Burn Pit / Smoke (7 Items)**	1. Heavy exposure to smoke or fumes from personally operating or working with trash incineration or at a burn pit? This includes regularly burning trash and burn pit security.<br>2. Heavy sustained exposure outdoors to smoke or fumes from burn pits or planned incineration, for example when the wind changed or there was other heavy exposure?<br>3. Heavy sustained exposure to smoke or fumes from burn pits or planned incineration coming into your work site or housing?<br>4. Heavy direct exposure to smoke or fumes from burning vehicles or fires caused by explosives or other weapons?<br>5. Heavy direct exposure to flares, marker smoke, smoke bombs, or other similar sources?<br>6. Other heavy exposure to smoke or fumes from other burning or open combustion sources not previously mentioned? Do not count engine exhaust.<br>7. Doing regular exercise or other physical exertion alongside of the perimeter or right beside a burn pit site?

© 2025 Hosseini R et al. *JAMA Network Open*.

eTable 2. Questionnaire Items Excluded Due to Low Factor Loading (<0.37) or Loading Across Multiple Factor Groups

1. Vehicle, aircraft, generator, or other engine refueling operations?
2. Welding or flame cutting?
3. Use of asbestos?
4. Use of fiberglass or insulation material other than asbestos?
5. Heavy sustained exposure from close proximity to truck, Humvee, tank, APC, or Stryker engine exhaust fumes?
6. Heavy exposure to any other engine or compressor exhaust fumes?
7. As a part of your duties was there any other regular heavy exposure to vapors, gas, dust, or fumes not already covered?
8. Other heavy exposure to smoke or fumes from other burning or open combustion sources not previously mentioned? Do not count engine exhaust.
9. Other pesticide, insecticide, or repellent application or handling?

eTable 3. Additional Participant Characteristics

**Income**	**No. (%)**
<$10k	23 (1.3)
$10k - <$30k	78 (4.6)
$30k - <$50k	212 (12.4)
$50k - <$75k	301 (17.6)
$75k - <$100k	327 (19.1)
≥$100k	718 (41.9)
**Marital status**	
Married	1,180 (68.9)
Never married	335 (19.6)
Divorced	250 (14.6)
Cohabiting	50 (2.9)
Separated	40 (2.3)
Widowed	8 (0.5)

© 2025 Hosseini R et al. *JAMA Network Open*.

eTable 4. Unadjusted (Crude) Odds Ratios for Respiratory Symptoms by Exposure Domain

Exposure Factor, No. (%) N = 1,712	Symptoms, No. (%)		
	Dyspnea 117/1672 (7.0%)	Wheeze 260/1712 (15.2%)	CB 121/1712 (7.1%)
	OR (95% CI)	OR (95% CI)	OR (95% CI)
**Combustion/Ground Dust, 1,014 (59.2)**	1.28 (1.02-1.60)	1.18 (1.01-1.39)	1.05 (0.83-1.33)
**Aircraft maintenance, 812 (47.4)**	1.02 (0.78-1.33)	1.19 (1.01-1.41)	1.05 (0.82-1.34)
**Heavy equipment maintenance, 783 (45.7)**	1.47 (1.21-1.79)	1.33 (1.15-1.54)	1.22 (1.00-1.49)
**Construction, 213 (12.4)**	1.08 (1.21-1.79)	0.89 (0.68-1.18)	1.01 (0.70-1.46)
**Incineration byproducts, 123 (7.2)**	1.03 (0.76-1.38)	1.07 (0.88-1.30)	1.05 (0.80-1.38)

Abbreviations: OR, odds ratio.

© 2025 Hosseini R et al. *JAMA Network Open*.

eTable 5. Adjusted Odds Ratios for Socioeconomic Covariates and Respiratory Symptoms

		**Dyspnea**	**Wheeze**	**Chronic Bronchitis**
		**OR (95% CI)**	**OR (95% CI)**	**OR (95% CI)**
**Income**	<$10k	1 (ref)	1 (ref)	1 (ref)
	$10k - <$30k	32.54 (0.79 - 4020.37)	3.82 (0.56 - 254.77)	0.07 (0.00 - 6.85)
	$30k - <$50k	85.94 (30.86 - >999.99)	5.28 (0.85 - 310.27)	0.43 (0.12 - 45.13)
	$50k - <$75k	51.66 (18.15 - >999.99)	3.23 (0.53 - 185.55)	0.51 (0.15 - 52.18)
	$75k - <$100k	54.08 (19.26 - >999.99)	4.29 (0.75 - 254.42)	0.45 (0.13 - 48.72)
	≥$100k	36.00 (12.80 - >999.99)	3.97 (0.72 - 234.82)	0.30 (0.09 - 34.65)
**Education**	High school diploma / GED	1 (ref)	1 (ref)	1 (ref)
	<high school completion	0.00 (0.00 - 0.01)	0.00 (0.00 - 0.01)	0.02 (0.00 - 0.09)
	Associate's degree	0.84 (0.39 - 1.96)	1.26 (0.70 - 2.39)	1.38 (0.64 - 3.65)
	Some college, but no degree	1.12 (0.58 - 2.54)	1.13 (0.66 - 2.00)	1.10 (0.50 - 2.85)
	Bachelor's degree	1.01 (0.51 - 2.31)	1.06 (0.61 - 1.98)	1.00 (0.46 - 2.55)
	Master's degree or higher	0.81 (0.33 - 2.15)	0.90 (0.44 - 1.82)	0.94 (0.39 - 2.27)

© 2025 Hosseini R et al. *JAMA Network Open*.
