# Supplementary material for: Military Inhalational Exposures Outside the Theater of Conflict and Chronic Respiratory Symptoms
Source: JAMA Netw Open. 2025 Jul 21;8(7):e2522080. doi: 10.1001/jamanetworkopen.2025.22080 (PMC12281241; doi:10.1001/jamanetworkopen.2025.22080)
Supplement: Supplement 2. — Data Sharing Statement [file jamanetwopen-e2522080-s002.pdf]

Data Sharing Statement

Hosseini. Military Inhalational Exposures Outside the Theater of Conflict and Chronic Respiratory Symptoms. *JAMA Netw Open*. Published July 21, 2025.
doi:10.1001/jamanetworkopen.2025.22080

Data
Data available: No
